# Rapid Identification and Simultaneous Quantification of Aristolochic Acids by HPLC-DAD and Confirmations by MS in *Aristolochia chilensis* Using a Limited Biomass

**DOI:** 10.1155/2018/5036542

**Published:** 2018-06-06

**Authors:** Michael Araya, Samantha García, Marcia González-Teuber

**Affiliations:** ^1^Centro de Investigación y Desarrollo Tecnológico de Algas (CIDTA), Facultad de Ciencias del Mar, Universidad Católica del Norte, Coquimbo, Chile; ^2^Departamento de Química, Facultad de Ciencias, Universidad de La Serena, La Serena, Chile; ^3^Laboratorio de Química Ecológica, Facultad de Química y Biología, Universidad de Santiago de Chile, Santiago, Chile; ^4^Instituto de Investigación Multidisciplinario en Ciencia y Tecnología, Universidad de La Serena, La Serena, Chile

## Abstract

Six aristolochic acids were identified in the Chilean species *Aristolochia chilensis* using high-performance liquid chromatography coupled to a diode array detector (HPLC-DAD) and subsequent confirmation with mass spectrometry (MS). The fractions of each signal were collected and injected directly into an Orbitrap mass detector model Q Exactive Focus (Thermo Scientific). The acids extraction was done with 0.10–0.50 g of lyophilized and pulverized sample and concentrated in Soxhlet extraction equipment. The liquid-liquid separations and a subsequent solid phase extraction (SPE) C18 were performed using 100 *µ*L of the extract that contains the aristolochic acids present in the *Aristolochia chilensis* plant. The HPLC conditions used a single mobile phase acetonitrile : water (1 : 1) acidified with 0.1% acetic acid and an isocratic elution to 1 mL·min^−1^. The column InertSustain C18 250 × 4.6 mm and 3 *µ*m was used, the injection volume was 20 *µ*L, and the time of run was reduced to 15 min. Calibration curves were constructed with *r* ^2^ being 0.9997. The quantification limit for AAI was 0.138 ± 0.010 *µ*g/mL, and for AAII, it was 0.558 ± 0.042 *µ*g/mL.

## 1. Introduction

The aristolochic acids (AAs) are derivatives of nitrophenanthrene present in plants of the genus *Asarum* spp. and *Aristolochia* spp. [1]. Contemporary medicine uses plant extracts for the therapy of arthritis, gout, rheumatism, and festering wounds [2], *Aristolochia* being used in obstetrics and snake bite treatments. The anti-inflammatory properties of AA enhance the development of pharmaceutical preparations; also, various studies have showed their strong carcinogen effect [1, 3, 4]. Despite the high variety of these acids, only seven have been described for *Aristolochia chilensis* [3, 5], focused in the AAI and AAII. These acids are responsible for their mutagenic and carcinogenic nature [3, 6]. The aristolochic acids can be detected and quantified by using HPLC-MS [4, 7, 8], IR, and NMR [9], but those are expensive methods. In the case of IR and NMR, they require a considerable quantity of the pure compound, which is very difficult to obtain when we have a small quantity of biomass. The technique of HPLC-DAD is considered one of the most suitable techniques for these analyses [10].

Methodologies in the analysis by HPLC reported in the literature used sample quantities ranging from 40 to 200 g [5, 11]. This represents a disadvantage because of the low plant abundance available, and it hinders sectoral studies. High variation in aristolochic acid concentrations has been described when the plant is subject to some mechanical stress or the action of the insect attack. In a practical way, the measurements are commonly performed with two or more mobile phases, greatly increasing the run times over 25 min [11, 12].

In the present work, we determined better conditions to extract and detect aristolochic acids from lower biomass samples. Changes in some parameters to improve the extraction are made, for example, using an Ultra-Turrax®, and the concentration of acids are determined by using Soxhlet extraction equipment. In addition, solid phase extraction was applied as a purification technique. These changes allow proper identification and quantification of AAs using limited amounts of plant biomass and in low concentrations, obtaining good resolution and well-defined chromatography signals.

## 2. Materials and Methods

### 2.1. Instrumentation

The measurements were carried in a Jasco HPLC-DAD model MD-2015 plus, controlled with the chrompass software. An InertSustain C18 column 250 × 4.6 mm, 3 *µ*m, was used as a stationary phase.

The correct order of elution was confirmed by MS. First, the sample was separated using HPLC-DAD, and full fraction collected was measured by direct injection in mass spectrometer Q Exactive focus with an Orbitrap® detector (Thermo Scientific).

### 2.2. Reagents

Each acid standard (AAI and AAII) was purchased separately from Sigma-Aldrich. HPLC-grade methanol and acetonitrile were purchased from Merck. The ultrapure water was prepared using the purification with the heal force model Smart Series. The other reagents such as chloroform, ethyl acetate, and methanol were of analytical quality and purchased from Merck.

### 2.3. Plant Material

*Aristolochia chilensis* samples were collected at different sites arranged in a rocky hillside in the supralittoral zone of Totoralillo beach (Coquimbo, Chile, 29° 58′ S; 71° 22′ W) [13, 14]. The samples were later transported to the laboratory of Centro de Investigación y Desarrollo Tecnológico en Algas (CIDTA) at Universidad Católica del Norte (Coquimbo), located 15 km away. All collected materials were stored at −80°C for 48 h and then lyophilized in freezer dryer Ilshin® TFD 8501 and ground for further analysis.

### 2.4. Standard Solution and Calibration Curves

Two standard solutions (1000 *µ*g/mL) of AAI and AAII acids were prepared by dissolving in methanol. The calibration curve was constructed according to Table 1.

The calibration curves were constructed for each aristolochic acid by plotting the peak area versus concentration of each standard. The limit of quantification was calculated for each one.

### 2.5. Sample Preparation

#### 2.5.1. Extraction of Aristolochic Acids

The extraction of aristolochic acids was carried weighing 0.4012 g of lyophilized and pulverized leaf of Aristolochia chilensis, 20 mL of methanol was added, and it was then homogenized with Ultra-Turrax for 2 min. The extract was transferred to Soxhlet equipment where the extraction continued for 2 hours at 50°C. The methanolic extract was evaporated to dryness in vacuum in a rotary evaporator Büchi R-300. The syrupy residue was suspended in 30 mL of 5% NaHCO_3_ and heated for 10 min at 40°C in a thermostatic bath. This solution was filtered by simple filtration using filter paper (MN 615 ∅ 125 mm) and transferred to a decanting funnel. The solution was washed with 15 mL (×3) of CHCl_3_, discarding the organic phase. The aqueous phase recovered was washed with 15 mL (×3) of ethyl acetate, discarding the higher yellow phase, followed by the addition of 1 N HCl until it reaches pH 2. Then, the AAs are extracted from the aqueous phase with 15 mL (×3) of ethyl acetate.

The three fractions of the ethyl acetate were mixed and filtered by adding a spatula of anhydrous sodium sulfate. From filtered solution, an aliquot of 10 mL was carried to dryness in vacuum. The residue was resuspended in 500 *µ*L of methanol HPLC-grade and 10 *µ*L of ammonia 1 N.

#### 2.5.2. Solid Phase Extraction of AA

The separation was performed on a C18 SPE column (Waters), conditioned with 1 mL of methanol HPLC-grade. Methanolic extract (100 *µ*L) was washed with 2 mL of ultrapure water. Subsequently, the aristolochic acid was eluted with 1.5 mL of acetonitrile acidified with 0.1% acetic acid, reserving the eluted fraction (pale yellow) for a later concentration. After that, 400 *µ*L from eluted extract was taken and brought to dryness under a stream of nitrogen gas. The residue was resuspended in 100 *µ*L of methanol HPLC-grade.

#### 2.5.3. Detection by HPLC-DAD

For HPLC-DAD detection, a mobile phase of ACN : H_2_O (1 : 1) acidified with 0.1% acetic acid was used and an isocratic flow to 1 mL·min^−1^. The oven temperature was 40°C, and 20 *µ*L of the sample was injected which was previously filtered through a 0.22 *µ*m membrane filter of polyvinylidene fluoride (PVDF). The detection was performed with a diode array detector (DAD), recording the spectra between 200 and 800 nm. The measurement was made at 254 nm. Each peak was collected using an automatic fraction collector (FC-2088-30, Loncotec).

#### 2.5.4. MS Analysis

Aristolochic acid detection was carried out in electrospray positive-ion mode [(+) ESI]. The measurements were recorded in full scan mode (scan range: 200–400 *μ*m, microscan: three scans per second. The ESI conditions were as follows: sheath gas flow rate: 12; aux gas flow rate: 0; sweep gas flow rate: 0; spray voltage: 3.5 kV; capillary temperature: 250°C; S-lens RF level: 100; aux gas heater temperature: 150°C. First, the collected fractions were measured in full scan mode and later MS/MS for the *m*/*z* 358, *m*/*z* 328, *m*/*z* 342, *m*/*z* 312, and *m*/*z* 372 ions was performed. The fragmentation was performed at CE 20 eV.

## 3. Results and Discussion

### 3.1. Choice of Mobile Phase

Usually, the aristolochic acid detection is performed using two or more phases which contain acetonitrile and acidified water [5, 15]. For this reason, we have tested this method using two phases: a water phase (phase A) acidified with acetic acid to 0.1% and second phase (phase B) acetonitrile acidified with 0.1% acetic acid. Both solutions were filtered (pore diameter 0.45 *µ*m) and sonicated (5 minutes). The gradient used begin of 0–5 min, with elution of 30% B; 5–45 min linear gradient from 30% B up to 45% B at a flow rate of 1 mL·min^−1^. Using this method, good separation was not obtained. The use of a single mobile phase has improved the performance of chromatography. The phase consists of a solvent mixture of 0.1% acetic acid in the ratio ACN : H_2_O (1 : 1), using an isocratic flow of 1 mL·min^−1^. An aliquot of 10 *µ*L of a mix of AAI and AAII standards was injected (Table 1) to corroborate the accuracy of measures.

### 3.2. Cleaning the Sample

The cleanliness of the sample is necessary because interferers exist in high concentrations which mask the analyte, with intense signals of up to approximately 2500 mAU.

**Figure 1:**
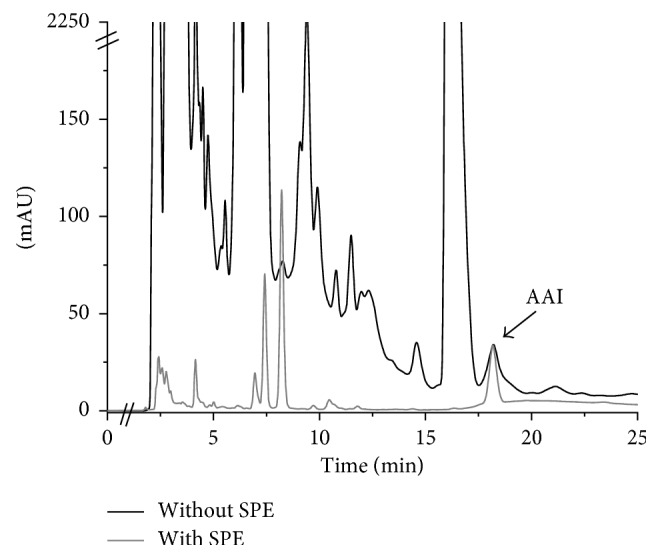
Comparison of signal between the extract of *Aristolochia chilensis* with and without the SPE column (measured in HPLC-DAD).

It can be observed (Figure 1) how the interferers are eliminated by applying the SPE column, which improves the separation and resolution of the extract chromatogram. Extract dilution is not recommended because the aristolochic acid signals are not detected.

### 3.3. Column Type: Particle Size and Carbon Load

Two columns were tested with different characteristics specified (Table 2).

For comparison, 10 *µ*L of standard mix AAI (200 *µ*g/mL) and AAII (800 *µ*g/mL) were injected with a single phase (see details in 3.1 Section).

**Figure 2:**
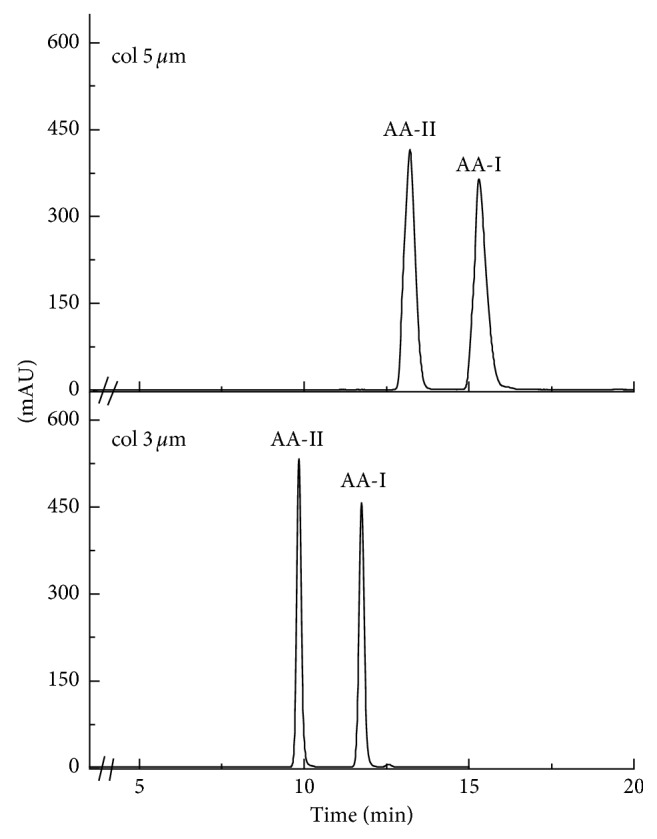
Comparison between columns for the measurement in the standard mix. Running conditions as in Section 2.5.3.

As can be observed in Figure 2, the use of a column with small particle diameter reduces the time of run. The mobile phase eluted slowly, increasing the pressure in the chromatographic system. In this condition, the sample increased its contact with the particles allowing the best extract separation without affecting the resolution. A difference of 2% less in the carbon charge causes the sample to be less retained, improving the elution time of about 5 min.

### 3.4. Identification of Aristolochic Acids

**Figure 3:**
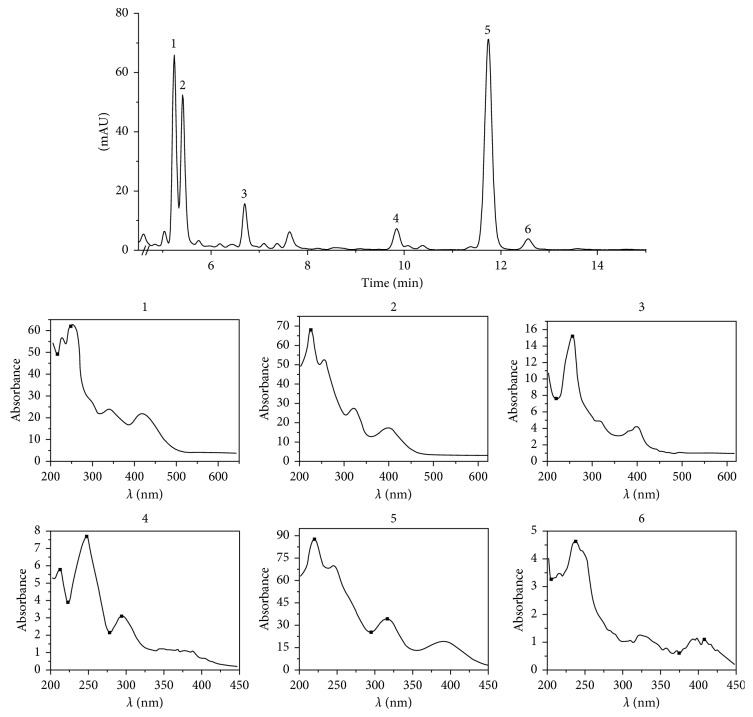
Chromatogram of the methanolic extract of *Aristolochia chilensis*, including the UV-Vis spectra for each acid detected: (1) AAIVa; (2) AAIa; (3) AAIII; (4) AAII; (5) AAI; (6) AAIV.

The spectra obtained were compared with the absorption spectra reported in the literature [5, 16], detecting six of the acids present in *Aristolochia chilensis* (Figure 3).

The commercial standards were used for AAI and AAII; therefore, for the others, only the percentage of relative abundance was calculated (Table 3).

**Figure 4:**
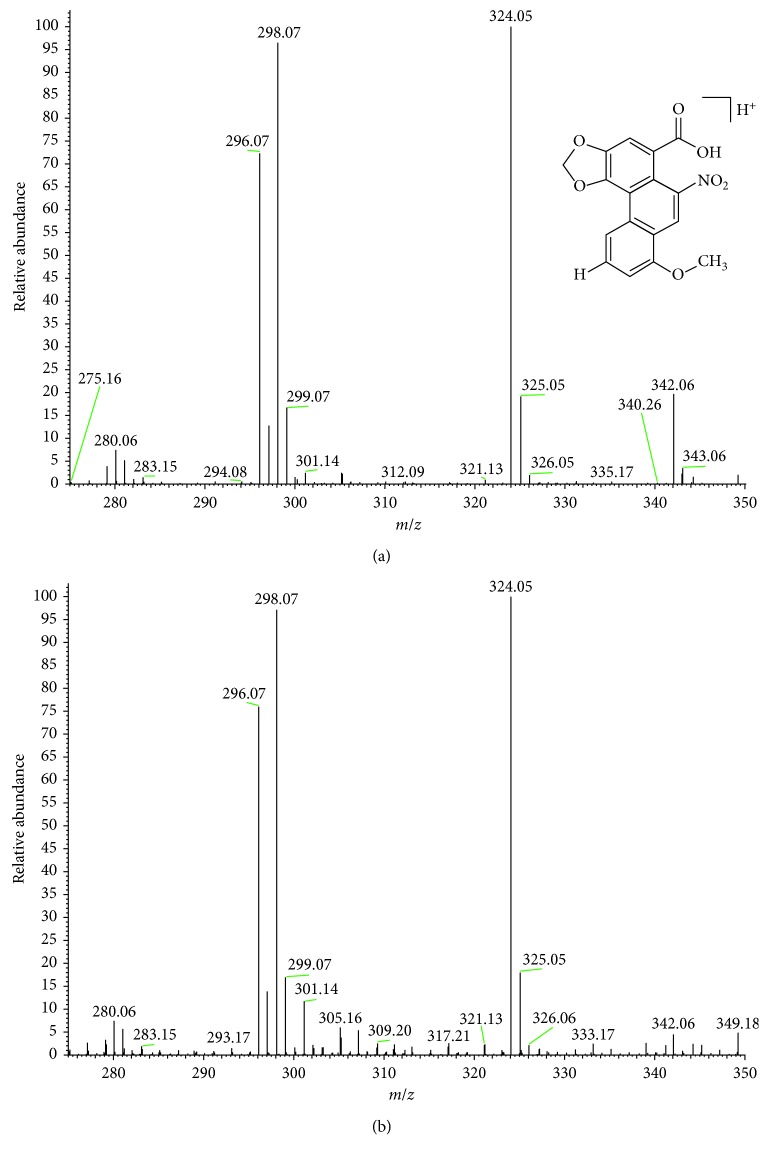
(a) Mass spectrum of standard AAI, full scan in positive-mode electrospray ionization. (b) Mass spectrum of collection the peak number 5 from the sample, corresponding to AAI, full scan in positive-mode electrospray ionization.

However, the absorption characteristics of some acids do not entirely coincide with the described characteristics. For example, the reported absorptions for IV acid are 244, 251, 285, 315, 364, and 393 [5] compared to 221, 242, 251, 293, 329, and 410 detected in the present study (Table 3). Some factors affecting the absorption characteristics include pH, mobile phase, and matrix, in addition to the low concentration making it difficult to obtain well-defined spectrum. To support the analysis, mass spectrum for each peak collected was obtained. For example, the full mass spectrum of the commercial standard AA I (Figure 4(a)) was compared with the full mass spectrum obtained for the collected peak 5 (Figure 4(b)). We concluded that the peak 5 collection corresponds to the AAI acid. The parent mass 342 and fragments 324, 298, and 396 were found (Table 4).

**Figure 5:**
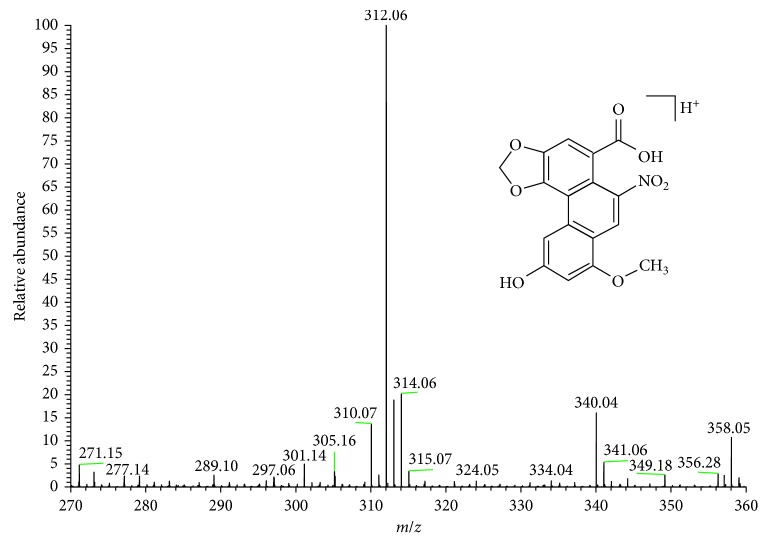
Mass spectrum of collection peak number 1. For these masses, AAIVa was assigned.

As an example, Figure 5 shows the full mass spectrum for peak number 1 collected. The parent mass 358 and fragments 312, 314, and 340 were found. These masses are assigned to the AAIVa acid.

The summary of masses and majority fragments for each peak and respective elution order are shown in Table 4. See Figures 8–11 in the supplementary material for comprehensive image analysis.

### 3.5. Quantification of AAI and AAII

**Figure 6:**
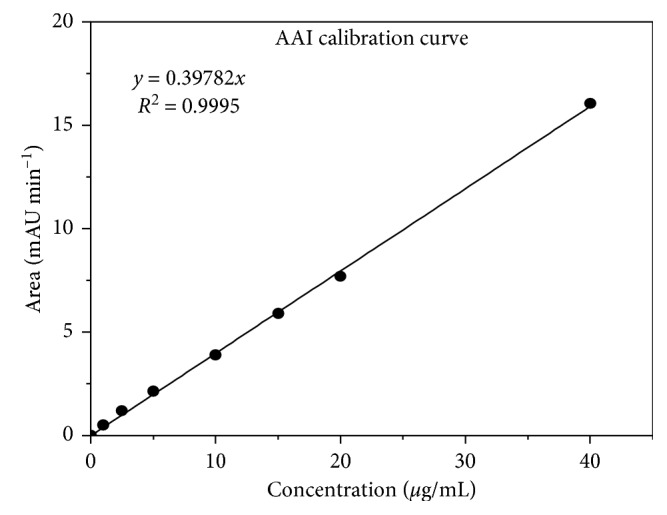
Calibration curve for AAI. LD AAI = 0.066 ± 0.010 *µ*g/mL and LQ AAI = 0.138 ± 0.010 *µ*g/mL.

**Figure 7:**
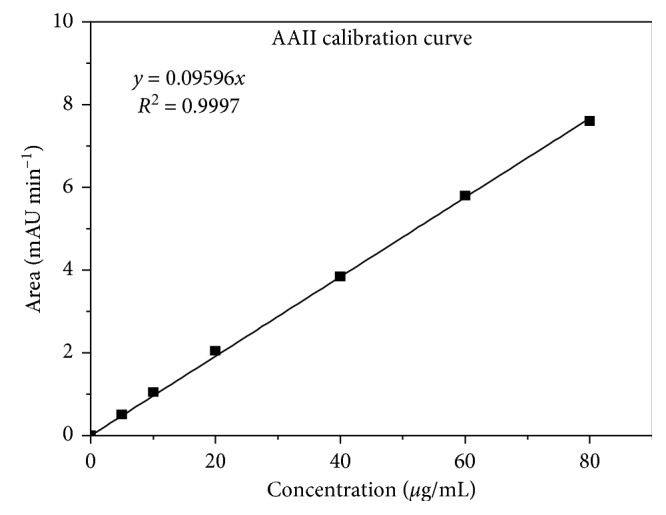
Calibration curve for AAII. LD AAII = 0.266 ± 0.042 *µ*g/mL and LQ AAII = 0.558 ± 0.042 *µ*g/mL.

The calibration curves for AA I (Figure 6) and AA II (Figure 7) were constructed, injecting 20 *μ*L of each standard mix (Table 1). Using the Chrompass software, each of the peaks was integrated to get the areas. The calibration curves were constructed plotting area versus concentration and applying a linear fit. Correlation coefficients with (*r* ^2^) ≥ 0.9997 were obtained, and limits of quantification (LQ) and detection (LD) for each acid (AAI and AAII) were included.

By interpolating the areas of each peak in Figures 6 and 7 for AAI and AAII, respectively, were found concentrations of 33.93 *µ*g/mL of AAI and 9.38 *µ*g/mL of AAII present in 20 *µ*L of injection.

The amount of aristolochic acid (*μ*g AA/g dry leaf) present in 0.4012 g of the original sample was calculated using the following equation:

(1) $$\begin{array}{c} \frac{\mu {\text{g}}_{\text{AA}}}{g_{\text{sample}}}=\left(\frac{C_{\mu \text{g}/\text{mL}}\times 0.1875\times V_{\text{acetate}}}{w_{\text{sample}}}\right), \end{array}$$

where 0.1875is the dilution factor, *V*_acetate_ is the mL ethyl acetate extract, and *w*_sample_ is the dry leaf sample weight.

The volume of ethyl acetate can be a variable according to the extraction. Considering a volume of 40 mL, the extract of *A. chilensis* contained 634.3 *µ*g/g dry of AAI and 175.3 *µ*g/g dry for AAII.

## 4. Conclusions

Methodology for identifying aristolochic acids present in *Aristolochia chilensis* has been defined and established. The limits of detection of 0.066 ± 0.010 *µ*g/mL and 0.266 ± 0.042 *µ*g/mL were determined for AA I and AA II, respectively. The difficulty in measuring these acids when the amount of biomass is limited (up to 0.1 g dry weight) was surpassed through the implementation of a C18 SPE columns and processes of preconcentration. This methodology could be used in ecological studies related to *A. chilensis* and plant-herbivore interaction. It is inferred that the methodology presented can be applied in other plant species with aristolochic acids that should be tested in future studies.

Since all AA standards or possible aristolactams present in the plant are not commercially available, it is only possible to identify each peak of the chromatogram by comparing it with the absorption spectra given in the literature; however, with these conditions, it was possible to confirm the presence of each acid using an MS detector and establishing its correct elution order.

## Figures and Tables

**Table 1:** Aristolochic acid concentration of standard solutions for the construction of the calibration curve.

Standard mix	AAI (*n*=4)		AAII (*n*=4)	
	*µ*g/mL	RSD (%)	*µ*g/mL	RSD (%)
1	1	0.02	5	0.01
2	2.5	0.28	10	0.07
3	5	0.07	20	0.07
4	10	0.14	40	0.21
5	15	0.14	60	0.14
6	20	0.14	80	0.14
7	40	0.07	160	0.05

**Table 2:** Characteristics of the columns tested.

Column C18	Dimensions (mm)	Particle size (*µ*m)	Carbon load (%)
Sunniest	4.6 × 250	5	16
InertSustain	4.6 × 250	3	14

**Table 3:** Relative percentage for each AA identified present in the extract of *Aristolochia chilensis*.

Peak number	AAs	Area (mAU/min)	RT (min)	UV-Vis *λ*_max_ (nm)	Relative %
1	AAIVa	6.4	5.23	221, 242, 251, 293, 329, 410	21.1
2	AAIa	5.7	5.41	224, 254, 320, 398	18.9
3	AAIII	1.9	6.69	251, 284, 314, 389	6.3
4	AAII	0.9	9.84	212, 251, 299	3.0
5	AAI	13.5	11.72	224, 251, 320, 395	44.7
6	AAIV	0.8	12.57	221, 242, 251, 293, 329, 410	2.6
Total area	30.2				

**Table 4:** Mass of the AAs and confirmatory fragments in *Aristolochia chilensis* extracts.

Peak number	AAs	Molecular weight	*m*/*z* ESI (+)
1	AAIVa	357	358.05 [M+H]^+^
			312.06 [M+H-H_2_O-OCH_3_]^+^
			314.06 [M+H-H_2_O-CO]^+^
			340.04 [M+H-H_2_O]^+^

2	AAIa	327	328.23 [M+H]^+^
			284.06 [M+H-CO_2_]^+^
			310.04 [M+H-H_2_O]^+^

3	AAIII	341	342.13 [M+H]^+^
			282.27 [M+H-CO_2_-CH_3_]^+^

4	AAII	311	312.36 [M+H]^+^
			284.33 [M+H-CO]^+^
			294.04 [M+H-H_2_O]^+^

5	AAI	341	342.06 [M+H]^+^
			296.07 [M+H-H_2_O-OCH_3_]^+^
			298.07 [M+H-H_2_O-CO]^+^
			324.05 [M+H-H_2_O]^+^

6	AAIV	371	372.12 [M+H]^+^
			328.06 [M+H-H_2_O-CO]^+^
			354.06 [M+H-H_2_O]^+^

## Supplementary Materials

Supplementary 1 Figure 8: mass spectrum of collection peak number 2. For these masses, AAIa was assigned.

Supplementary 2 Figure 9: mass spectrum of collection peak number 3. For these masses, AAIII was assigned.

Supplementary 3 Figure 10: mass spectrum of collection peak number 4. For these masses, AAII was assigned.

Supplementary 4 Figure 11: mass spectrum of collection peak number 6. For these masses, AAIV was assigned.

## References

[1] Jordan S. A., Pewaiz S., Wexler P. (2014). Aristolochic acid. *Encyclopedia of Toxicology*.

[2] Balachandran P., Wei F., Lin R.-C., Khan I. A., Pasco D. S. (2005). Structure activity relationships of aristolochic acid analogues: toxicity in cultured renal epithelial cells. *Kidney International*.

[3] Michl J., Ingrouille M. J., Simmonds M. S. J., Heinrich M. (2014). Naturally occurring aristolochic acid analogues and their toxicities. *Natural Product Reports*.

[4] Chan W., Cui L., Xu G., Cai Z. (2006). Study of the metabolism of aristolochic nephrotoxin in phase I and phase II by liquid chromatography/tandem mass spectrometry. *Rapid Communications in Mass Spectrometry*.

[5] Urzúa A., Olguín A., Santander R. (2013). Aristolochic acids in the roots of *Aristolochia chilensis*, a dangerous chilean medicinal plant. *Journal of the Chilean Chemical Society*.

[6] Debelle F. D., Vanherweghem J.-L., Nortier J. L. (2008). Aristolochic acid nephropathy: a worldwide problem. *Kidney International*.

[7] Yu J., Ma C.-M., Wang X. (2016). Analysis of aristolochic acids, aristololactams and their analogues using liquid chromatography tandem mass spectrometry. *Chinese Journal of Natural Medicines*.

[8] Lee M. C., Tsao C. H., Lou S. C., Chuang W. C., Sheu S. J. (2003). Analysis of aristolochic acids in herbal medicines by LC/UV and LC/MS. *Journal of Separation Science*.

[9] Pradeepa V., Sathish-Narayanan S., Kirubakaran S. A., Thanigaivel A., Senthil-Nathan S. (2015). Toxicity of aristolochic acids isolated from *Aristolochia indica* Linn (Aristolochiaceae) against the malarial vector *Anopheles stephensi* Liston (Diptera: Culicidae). *Experimental Parasitology*.

[10] Lee T.-Y., Wu M.-L., Deng J.-F., Hwang D.-F. (2002). High-performance liquid chromatographic determination for aristolochic acid in medicinal plants and slimming products. *Journal of Chromatography B*.

[11] Urzúa A., Santander R., Sotes G. (2009). Aristolochic acids from *Aristolochia bridgesii*, a host-plant of battus polydamas archidamas. *Journal of the Chilean Chemical Society*.

[12] Yuan J., Liu Q., Zhu W., Ding L., Tang F., Yao S. (2008). Simultaneous analysis of six aristolochic acids and five aristolactams in herbal plants and their preparations by high-performance liquid chromatography-diode array detection-fluorescence detection. *Journal of Chromatography A*.

[13] Di Castri F., Hajek E. (1976). *Bioclimatología de Chile*.

[14] Stotz G. C., Gianoli E. (2013). Pollination biology and floral longevity of *Aristolochia chilensis* in an arid ecosystem. *Plant Ecology and Diversity*.

[15] Santander R., Urzúa A., Olguin A., Sanchez M. (2015). Temporal variation of *Aristolochia Chilensis* aristolochic acids during spring. *Molecules*.

[16] Zhang C., Wang X., Shang M. (2006). Simultaneous determination of five aristolochic acids and two aristololactams in aristolochia plants by high-performance liquid chromatography. *Biomedical Chromatography*.

