# Supplementary figures and images for: Rapid Identification and Simultaneous Quantification of Aristolochic Acids by HPLC-DAD and Confirmations by MS in Aristolochia chilensis Using a Limited Biomass
Source: J Anal Methods Chem. 2018 Jun 6;2018:5036542. doi: 10.1155/2018/5036542 (PMC6011054; doi:10.1155/2018/5036542)

## Slide 1
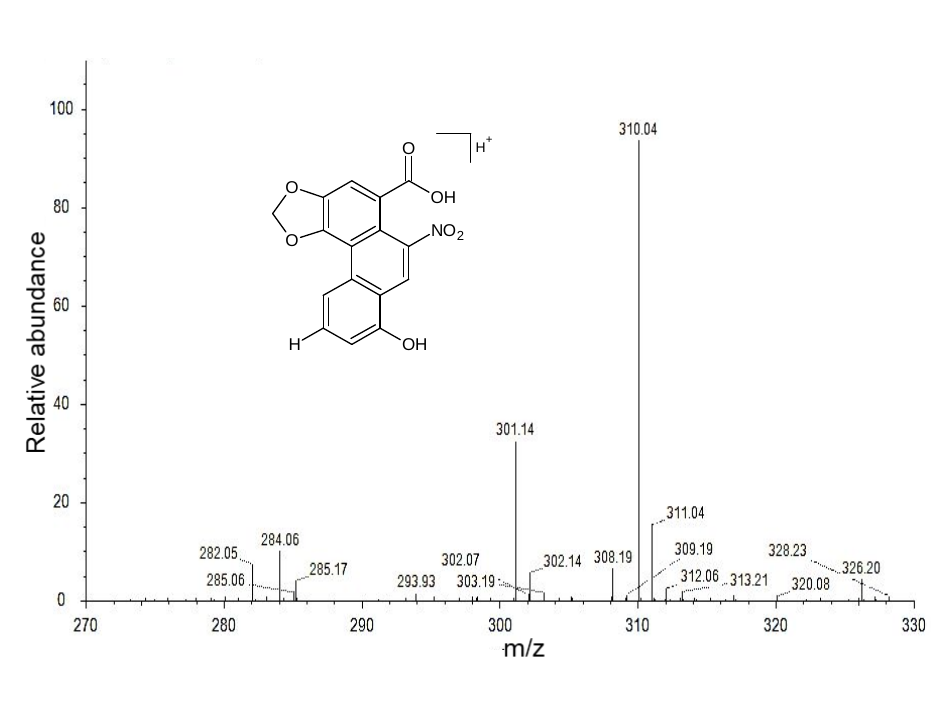

Supplement: Supplementary 1 — Figure 8: mass spectrum of collection peak number 2. For these masses, AAIa was assigned. [file 5036542.f1.pptx]

## Slide 1
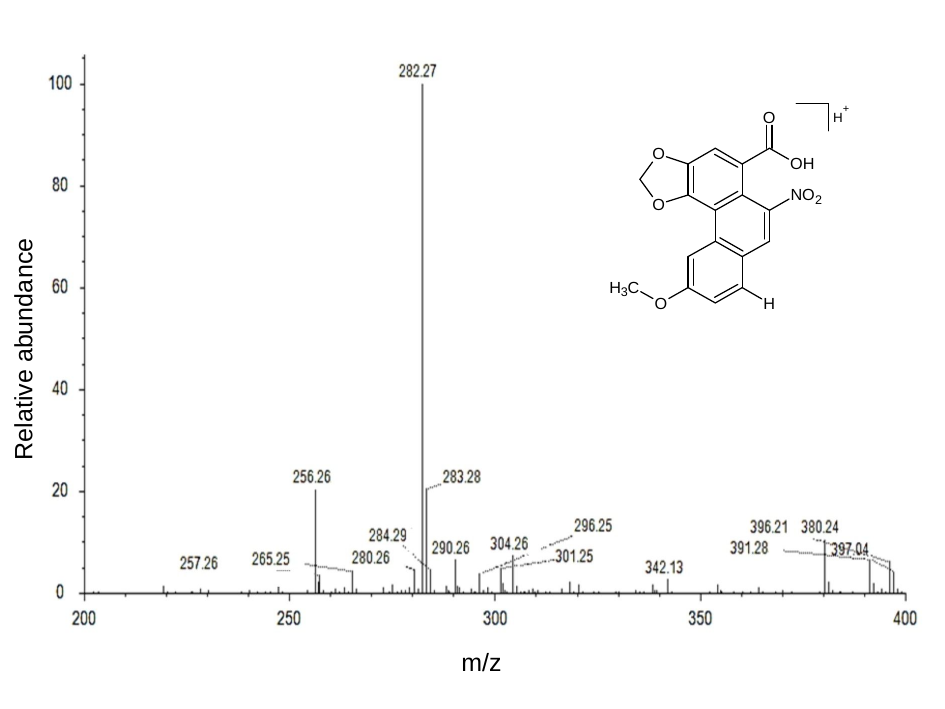

Relative abundance
m/z

Supplement: Supplementary 2 — Figure 9: mass spectrum of collection peak number 3. For these masses, AAIII was assigned. [file 5036542.f2.pptx]

## Slide 1
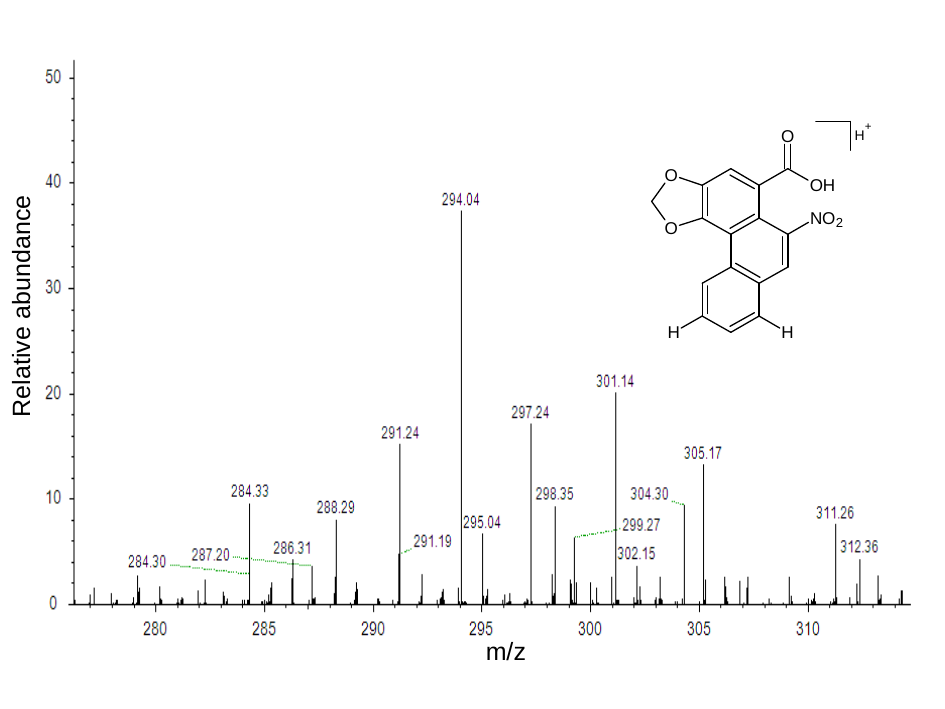

Relative abundance
m/z

Supplement: Supplementary 3 — Figure 10: mass spectrum of collection peak number 4. For these masses, AAII was assigned. [file 5036542.f3.pptx]

## Slide 1
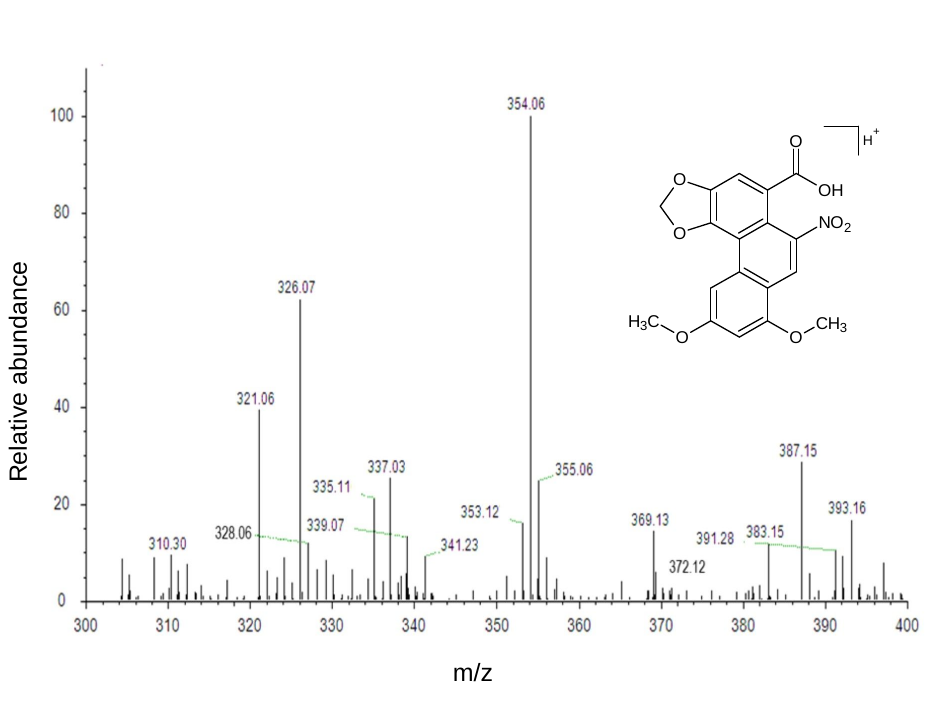

Relative abundance
m/z

Supplement: Supplementary 4 — Figure 11: mass spectrum of collection peak number 6. For these masses, AAIV was assigned. [file 5036542.f4.pptx]
